# A twenty gene-based gene set variation score reflects the pathological progression from cirrhosis to hepatocellular carcinoma

**DOI:** 10.18632/aging.102518

**Published:** 2019-12-15

**Authors:** Yan Lin, Rong Liang, Jiazhou Ye, Qian Li, Ziyu Liu, Xing Gao, Xuemin Piao, Rongyun Mai, Lianying Ge, Donghua Zou

**Affiliations:** 1 Department of Medical Oncology, Affiliated Tumor Hospital of Guangxi Medical University, Nanning 530021, Guangxi, People’s Republic of China; 2 Department of Hepatobiliary Surgery, Affiliated Tumor Hospital of Guangxi Medical University, Nanning 530021, Guangxi, People’s Republic of China; 3 Department of Endoscopy, Affiliated Tumor Hospital of Guangxi Medical University, Nanning 530021, Guangxi, People’s Republic of China; 4 The Fifth Affiliated Hospital of Guangxi Medical University, Nanning 530021, Guangxi, People’s Republic of China; * Equal contribution

**Keywords:** hepatocellular carcinoma, gene set, multistep hepatocarcinogenesis, HCC

## Abstract

The molecular mechanism of the pathological progression from cirrhosis to hepatocellular carcinoma (HCC) remains elusive. In the present study, tissue samples from normal liver, cirrhosis and HCC were subjected to differentially gene expression analysis, weighted gene correlation network analysis to identify the twenty hub genes (TOP2A, CDC20, PTTG1, CDCA5, CCNB2, PRC1, KIF20A, SF3B4, HSP90AB1, FOXD2, PLOD3, CCT3, SETDB1, VPS45, SPDL1, RACGAP1, MED24, KIAA0101, ZNF282, and USP21) in the pathological progression from cirrhosis to HCC. Each sample was calculated a hub gene set variation analysis (HGSVA) score using Gene Set Variation Analysis, The HGSVA score significantly increased with progression from cirrhosis to HCC, and this result was validated in two independent data sets. Moreover, this score may be used as a blood-based marker for HCC and is an independent prognostic factor of recurrence-free survival (RFS) and overall survival (OS). High expression of the hub genes may be driven by hypomethylation. The twenty gene-based gene set variation score may reflect the pathological progression from cirrhosis to HCC and is an independent prognostic factor for both OS and RFS.

## INTRODUCTION

Hepatocellular carcinoma (HCC) is the most common type of primary liver cancer and the third leading cause of cancer-related death worldwide [1]. Its clinical characteristics show a gradually increasing incidence and a poor prognosis. The recurrence rate is 42.9% within two years after curative treatment [2]. Although many drugs have been tested or under exploration [3–6], none have been confirmed to increase recurrence-free survival (RFS). Therefore, it is very important to study the pathological mechanism of HCC.

The incidence of HCC, which can be caused by various factors, varies widely worldwide, and this disease is often associated with chronic HBV or HCV infection. The pathogenesis of HCC is very complex, comprising multiple genetic and epigenetic alterations, chromosomal aberrations, gene mutations, and altered molecular pathways [7]. Chronic liver injury, inflammation, hepatocyte degeneration/regeneration, cirrhosis, necrosis and small cell dysplasia can be observed during multistep hepatocarcinogenesis [8]. There are various causes of cirrhosis, such as excessive alcohol consumption, contact with or consumption of Aspergillus toxins, and various metabolic disorders [9], but the mechanism of progression from cirrhosis to HCC remains elusive. Therefore, there is an urgent need to identify the hub gene or gene set dictating the progression from cirrhosis to HCC and to develop potential therapeutic targets.

In the present study, twenty hub genes were identified by WGCNA. The hub gene set variation analysis (HGSVA) score increased significantly with the progression from cirrhosis to HCC and was validated in two independent data sets. In addition, the HGSVA score is an independent prognostic factor for overall survival (OS) and RFS.

## RESULTS

### Differentially expressed genes (DEGs) in cirrhosis and HCC

**Figure 1:**
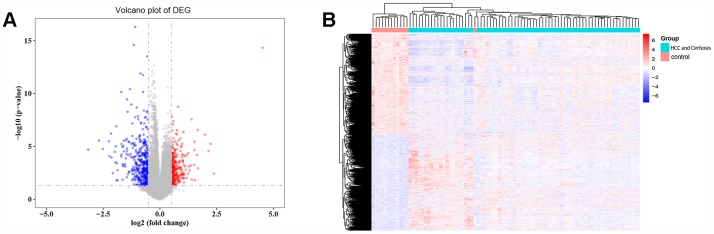
**Differentially expressed gene (DEG) analysis.** (**A**) Volcano plot of the DEGs. Red indicates upregulated and blue indicates downregulated in cirrhosis/HCC. (**B**) Hierarchical clustering dendrograms of the expression patterns of DEGs that can basically distinguish normal liver tissue and cirrhosis/HCC.

A total of 3525 DEGs, 1793 downregulated and 1732 upregulated, were identified in cirrhosis and HCC samples compared to control samples (Figure 1A). Cluster analysis showed that these DEG patterns could basically distinguish cirrhosis and HCC tissues from normal liver tissues (Figure 1B).

### Module associated with the progression from cirrhosis to HCC

**Figure 2:**
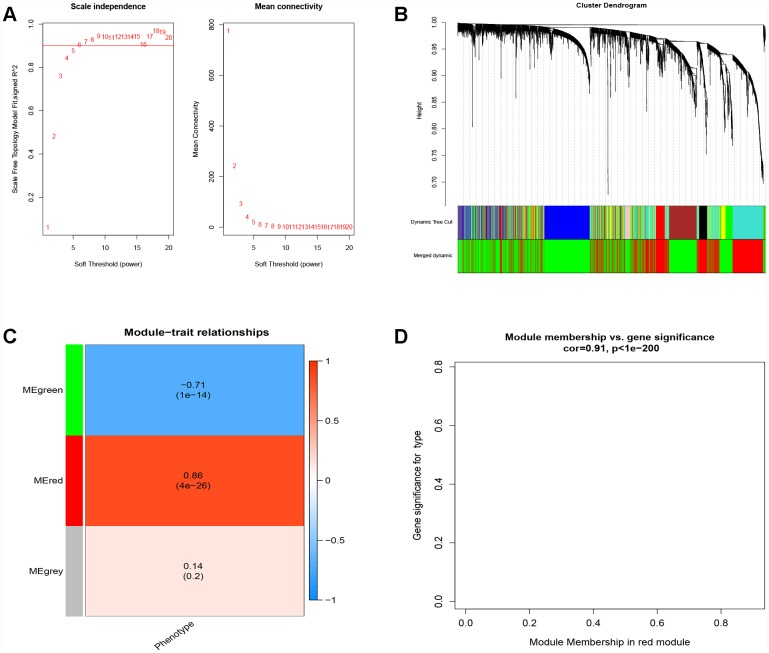
**Weighted correlation network analysis.** (**A**) Define power related to modules. (**B**) Recognition module. (**C**) The red module is positively correlated with phenotype, the green module is negatively correlated with phenotype, and gray is not related to phenotype. (**D**) MM and GS are highly correlated in the red module.

To find the key module most associated with the progression from cirrhosis to HCC, WGCNA was performed using the expression profile of the DEGs. The power was 7, which was the lowest value for the scale with an independence degree of up to 0.90 (Figure 2A). Three modules were identified (Figure 2B). The red module was positively correlated with phenotype (correlation coefficient = 0.86, P = 4E-26; Figure 2C), while the green module was negatively correlated with phenotype (correlation coefficient = -0.71, P = 1E-14; Figure 2C). This finding indicated that with the progression from cirrhosis to HCC, the expression patterns of the red module genes showed an increasing trend, while those of the green module genes showed a decreasing trend. The red module was selected as our candidate module in which to identify hub genes. The module membership (MM) and gene significance (GS) were highly associated in the red module (Figure 2D).

### Key pathways in the progression from cirrhosis to HCC

**Figure 3:**
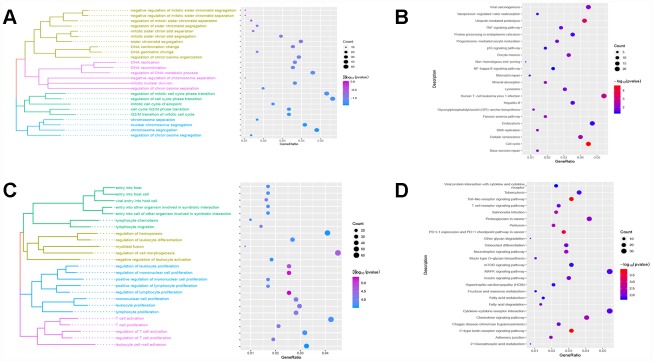
**Results of functional enrichment.** (**A**) The red module is significantly enriched in biological processes. (**B**) The red module is significantly enriched in KEGG pathways. (**C**) The green module is significantly enriched in biological processes. (**D**) The green module is significantly enriched in KEGG pathways.

Red module genes were significantly involved in biological processes related to cell proliferation and differentiation (Figure 3A) and in cirrhosis- or HCC-related pathways, such as hepatitis B and the TNF and P53 signaling pathways (Figure 3B). Green module genes were significantly enriched in biological processes related to T cells, leukocytes and lymphocytes (Figure 3C) and in the MAPK signaling pathway (Figure 3D).

### The HGSVA score significantly increased with the progression from cirrhosis to HCC

**Figure 4:**
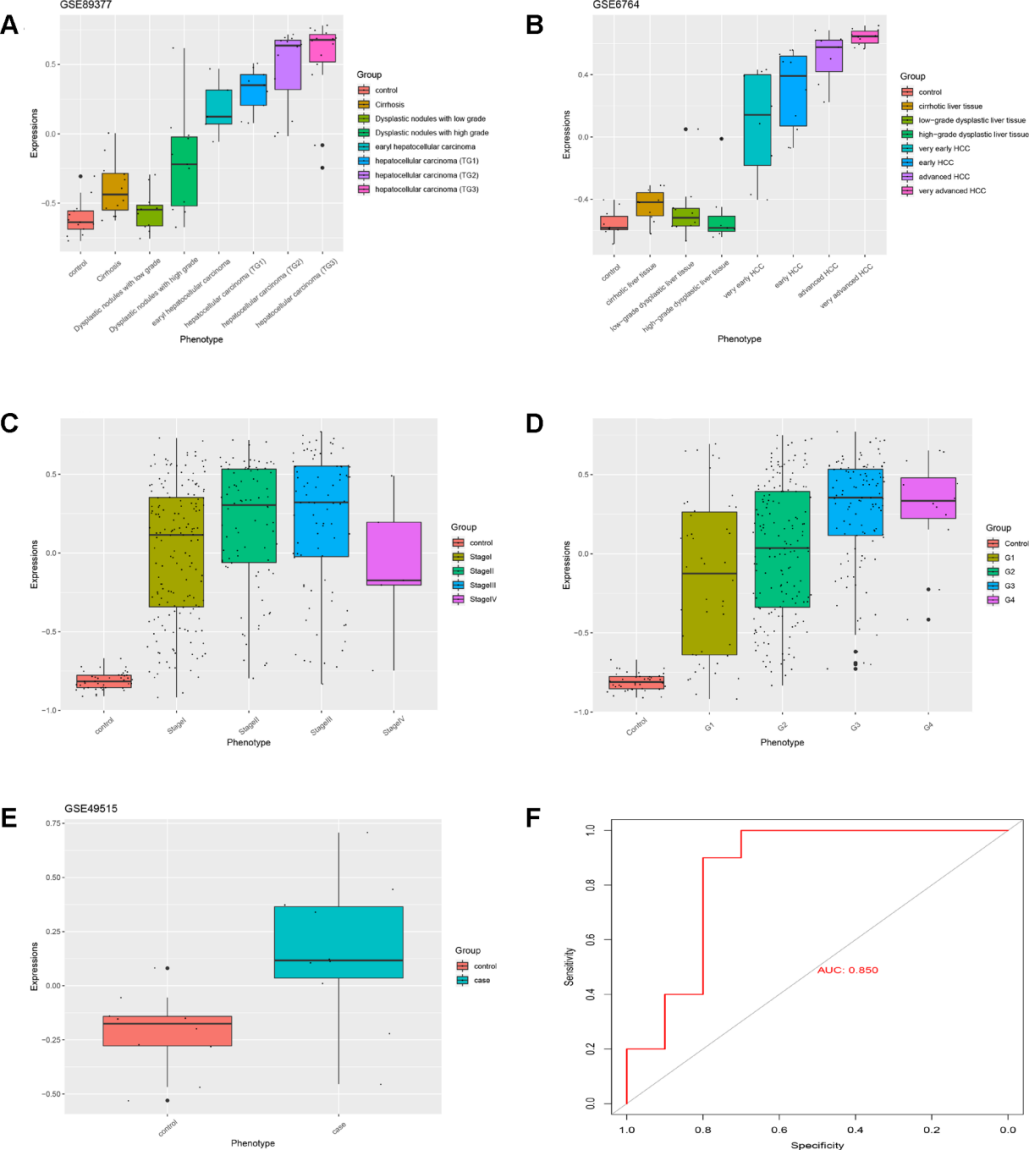
**The HGSVA score increased significantly with the progression from cirrhosis to HCC.** (**A**) The HGSVA score in GSE89377. (**B**) The HGSVA score in GSE6764. (**C**) The HGSVA score in the TCGA HCC data set (phenotype defined by pathological stage). (**D**) The HGSVA score in the TCGA HCC data set (phenotype defined by tissue grade). (**E**) The HGSVA score in GSE6764. (**F**) ROC results: the AUC is significant, which indicates that the gene set can be used for the early diagnosis of HCC.

Based on GS > 0.7 and MM > 0.8, twenty genes (TOP2A, CDC20, PTTG1, CDCA5, CCNB2, PRC1, KIF20A, SF3B4, HSP90AB1, FOXD2, PLOD3, CCT3, SETDB1, VPS45, SPDL1, RACGAP1, MED24, KIAA0101, ZNF282, and USP21) were identified as hub genes. The HGSVA score significantly increased with the progression from cirrhosis to HCC in GSE89377 (Figure 4A), and this finding was validated in the GSE6764 (Figure 4B) and TCGA HCC data sets (Figure 4C, 4D). This increasing trend was more pronounced in HCC.

### The HGSVA score may be a blood-based marker and independent predictor of OS and RFS in HCC

**Figure 5:**
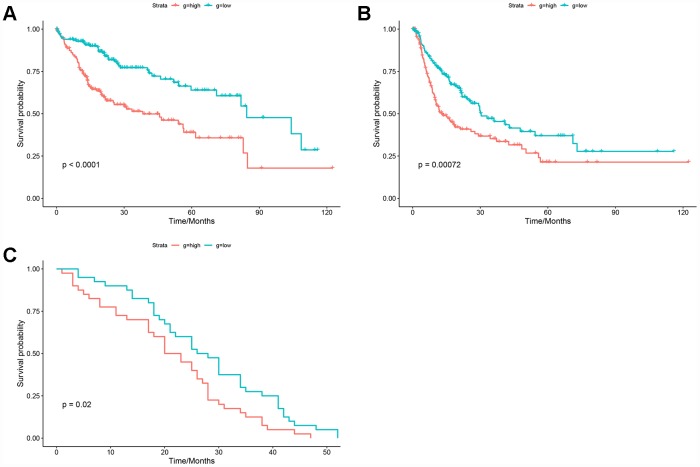
**Prognostic value of the HGSVA score.** (**A**) The HGSVA score was associated with overall survival. (**B**) The HGSVA score was associated with recurrence-free survival. (**C**) The HGSVA score was associated with overall survival, which was verified by the whole blood profile.

The HGSVA score in peripheral blood samples was significantly higher for patients with HCC than for healthy controls (Figure 4E). ROC curve analysis showed that the HGSVA score has potential as a blood-based marker for HCC (AUC=0.850, Figure 4F). The HGSVA score was significantly associated with the OS (Figure 5A) and RFS of patients with HCC (Figure 5B). This result was consistent with that for GSE49515 (Figure 5C). Among patients with HCC, those in the high HGSVA score group had a shorter OS and RFS. than those in the low score group. Furthermore, univariate and multivariate Cox analyses showed that the HGSVA score was a significantly independent factor for both OS (Table 1) and RFS (Table 2) after adjusting for routine clinicopathological features

**Table 1:** Univariate/Multivariate cox analyses of overall survival.

**Factor**	**Univariate Cox analysis**			**Multivariate Cox analysis**		
	**b**	**P**	**HR (95% CI)**	**b**	**P**	**HR (95% CI)**
Gender (male/female)	-0.232	0.228	0.544-1.156			
Age (>65 years/<=65 years)	0.239	0.202	0.88-1.833			
Grade (G3-4/G1-2)	0.131	0.498	0.781-1.663			
T stage (T3-4/T1-2)	0.919	0.000	1.723-3.649	0.348	0.733	0.192-10.457
Lymph node stage (N2-3/N0-1)	0.684	0.341	0.485-8.086			
Metastasis(M1/M0)	1.382	0.019	1.252-12.679	0.789	0.195	0.667-7.263
Pathological stage (III-IV/I-II)	0.911	0.000	1.711-3.613	0.589	0.562	0.246-13.23
HGSVA score (high/low)	0.864	0.000	1.614-3.488	1.069	0.000	1.663-5.098

HR, Hazard Ratio; CI, confidence interval; HGSVA, hub gene set variation analysis.

**Table 2:** Univariate/Multivariate cox analyses of recurrence-free survival.

**Factor**	**Cox analysis**			**Multivariate Cox analysis**		
	**b**	**P**	**HR(95% CI)**	**b**	**P**	**HR(95% CI)**
Gende r(male/female)	-0.009	0.957	0.71-1.384			
Age (>65 years/<=65 years)	-0.030	0.854	0.709-1.329			
Grade (G3-4/G1-2)	0.101	0.538	0.803-1.524			
T stage (T3-4/T1-2)	0.887	0.000	1.735-3.400	0.708	0.484	0.28-14.746
Lymph node stage (N2-3/N0-1)	0.343	0.631	0.348-5.715			
Metastasis (M1/M0)	0.909	0.206	0.607-10.150			
Pathological stage (III-IV/I-II)	0.865	0.000	1.7-3.3200	0.121	0.904	0.157-8.107
HGSVA score (high/low)	0.554	0.001	1.269-2.387	0.474	0.004	1.168-2.211

HR, Hazard Ratio; CI, confidence interval; HGSVA, hub gene set variation analysis.

### Hub genes are also highly expressed at the protein level in HCC

**Figure 6:**
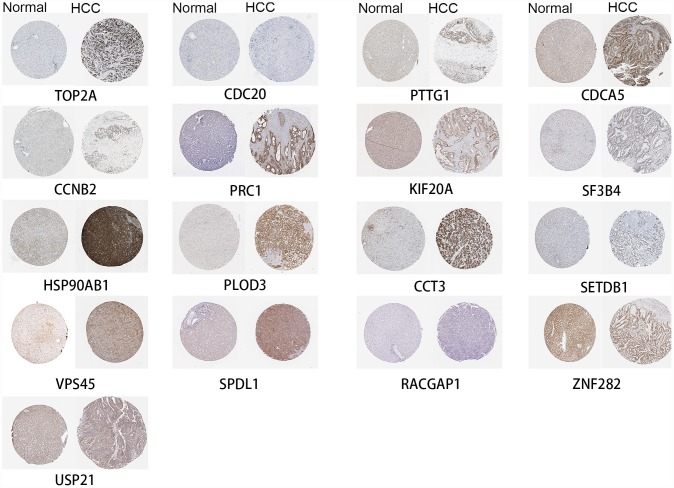
**High expression of genes by immunohistochemistry.**

Seventeen identified genes are included in The Human Protein Atlas, and all were highly expressed in HCC compared to normal liver (Figure 6). The antibodies against these proteins are listed in Supplementary Table 1. These data provide strong support for our results. FOXD2, MED24 and KIAA0101 are not in The Human Protein Atlas.

### The upregulation of hub genes by hypomethylation

**Figure 7:**
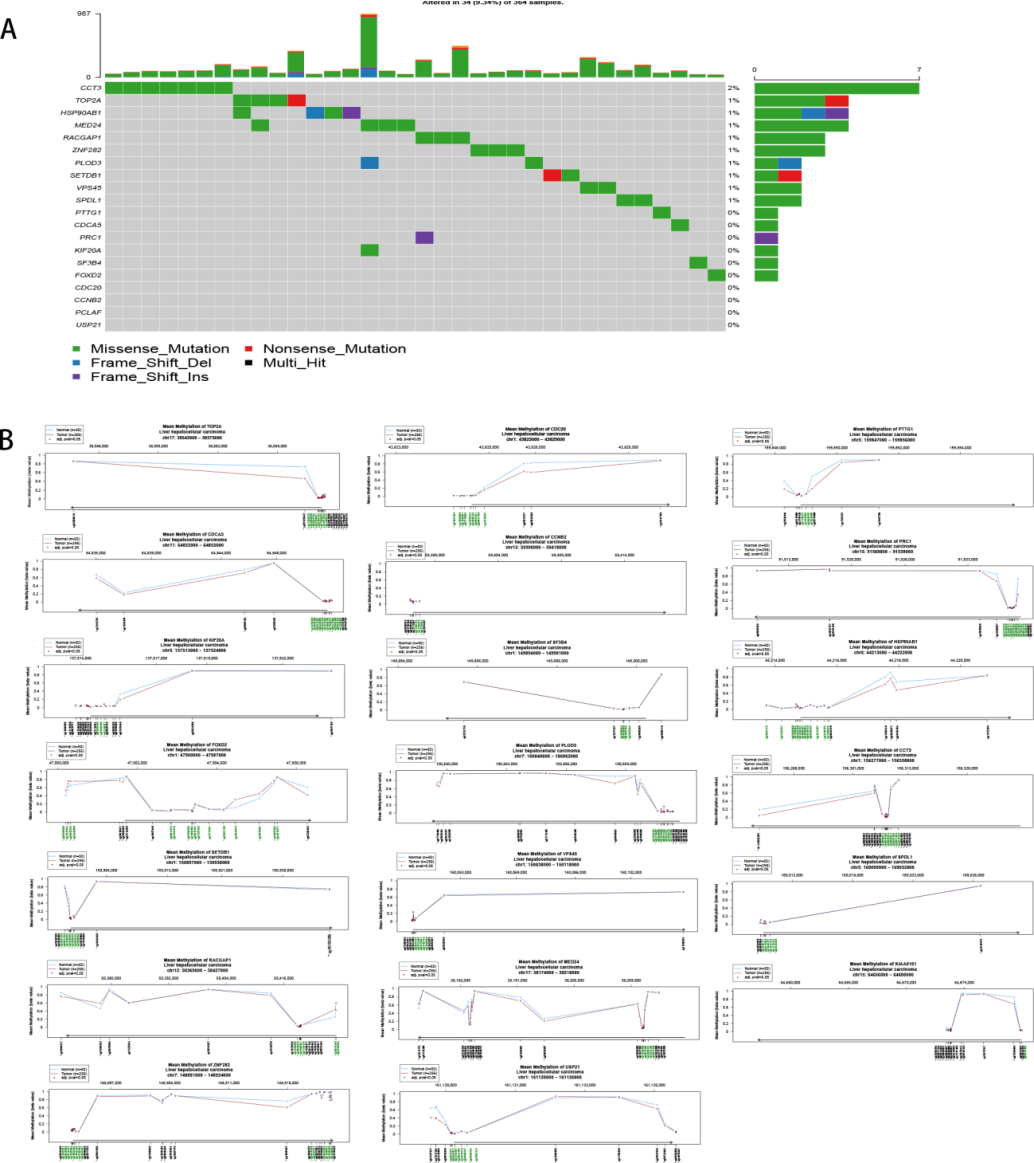
(**A**) Mutation of the 20 hub genes. (**B**) Methylation of the 20 hub genes.

The 20 hub genes were scanned for genetic alterations (Figure 7A), and a few samples had mutations (9.34%). This finding indicated that mutation may not be the cause of the differential gene expression. Compared to normal liver tissue, HCC tissue harbored one or more differentially methylated sites in the 20 hub genes; therefore, the upregulation of the hub genes was driven by hypomethylation (Figure 7B).

## DISCUSSION

Cirrhosis of any etiology increases the risk of HCC [10–12]. The sequential progression of cirrhosis to HCC is characterized by nodular lesions, such as low- and high-grade dysplastic nodules, which progress to early HCC and then to intermediate/advanced HCC. The slow progression from cirrhosis to HCC may be the key to preventing HCC, but the mechanism underlying this progression is not well understood. In our present study, we screened DEGs between cirrhosis/HCC and normal liver to perform WGCNA. Two functional modules were identified: one module (red module) was significantly positively correlated with the progression from cirrhosis to HCC, and the other module (green module) was significantly negatively correlated with this progression. This result suggests that liver disease progression is driven by multiple genes rather than a single gene. The red module genes were significantly involved in several cancer-related pathways, such as the cell cycle and the TNF and P53 signaling pathways, and in viral carcinogenesis and the hepatitis B pathway. HBV and HCV are the main viral pathogenic factors of cirrhosis and HCC.

Previous studies have focused on the role of a single gene or molecule. In the present study, twenty genes were identified as hub genes. Unsurprisingly, some have been associated with HCC, such as CD20 [13], PTTG1 [14], CDCA5 [15], HSP90AB1 [16], CCT3 [17], SF3B4 [16], SETDB1 [17], VPS45 [18], RACGAP1 [19], USP21 [20, 21], and KIF20A [22]. In addition, we also found that MED24, FOXD2 and ZNF282 are associated with the progression from cirrhosis to HCC. However, no single molecule is currently widely identified or targeted in the clinic, perhaps due to the heterogeneity of HCC. Thus, we used GSVA to score a sample rather than a gene (molecule). The HGSVA score significantly increased with the progression from cirrhosis to HCC and was validated in two independent data sets. This increasing trend was more pronounced upon the formation of HCC. Notably, the trend was not obvious before HCC formation in GSE6764, potentially because of the etiology of HCV in GSE6764. This finding indicates that the pathological progression from cirrhosis to HCC is complex and related to the etiology.

ROC analysis revealed that the HGSVA score had a high AUC (0.850) and that it may be a blood-based marker for HCC. This score may help diagnose patients with ambiguous imaging results. The HGSVA score was associated with prognosis; a higher score indicated a shorter OS and RFS. The results of the univariate and multivariate Cox regression analyses showed that the HGSVA score is an independent prognostic factor after adjusting for routine clinicopathological features. Surveillance is cost-effective and dictated by the incidence of HCC [23, 24]. High-score patients may be followed up more frequently than low-score patients based on the prognostic stratification system.

We identified mutations in the 20 hub genes in a few patients. Most of the genes had low methylation levels. Aberrant DNA methylation plays a crucial role in carcinogenesis [25, 26]. Therefore, we speculate that hypomethylation may be one cause of the upregulation of the 20 hub genes. The specific mechanism should be further explored, and further experimental verification is needed.

Herein, we provide new insights into the progression from cirrhosis to HCC. This study may represent the first report of a score for cirrhosis and HCC samples using a hub gene set. However, several limitations of the present study should be noted. First, the molecular mechanisms of these 20 genes in multistep hepatocarcinogenesis are not yet clearly understood, and it is unknown whether HGSVA is a causal factor or merely a marker of the pathological progression from cirrhosis to HCC. Second, the HGSVA score still needs to be confirmed in prospective trials before clinical application. Third, GSVA is more expensive, so further cost reductions are necessary before it can be applied in the clinic. In conclusion, we identified and validated a hub gene set in the pathological progression from cirrhosis to HCC. The twenty gene-based gene set variation score reflects the pathological progression from cirrhosis to HCC and is an independent prognostic factor for both OS and RFS.

## MATERIALS AND METHODS

### Data collection and processing

**Figure 8:**
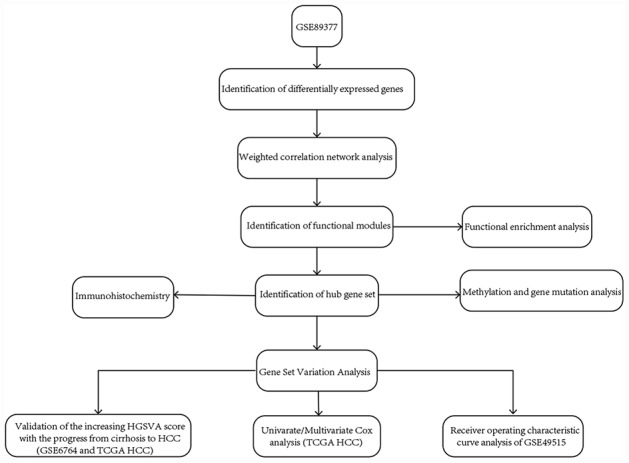
**The workflow of the present study.**

We downloaded the hepatic tissue gene expression profiles in GSE89377 from the GEO website (https://www.ncbi.nlm.nih.gov/) [27]; this dataset includes 13 normal liver, 8 low-grade chronic hepatitis, 12 high-grade chronic hepatitis, 12 cirrhosis, 11 low-grade dysplastic nodules, 11 high-grade dysplastic nodules, 5 early HCC, 9 grade 1 HCC, 12 grade 2 HCC and 14 grade 3 HCC. GSE89377 is based on GPL6947. The gene expression profiles in GSE6764 [28] are based on GPL570, including 10 normal controls, 10 cirrhotic liver tissues, 3 cirrhotic liver tissues from patients without HCC, 9 low-grade dysplastic liver tissues, 7 high-grade dysplastic liver tissues, 8 very early HCC, 10 early HCC, 6 advanced HCC, and 9 very advanced HCC. The whole blood gene expression profiles in GSE49515 [29] are based on GPL570, including 10 HCC and 10 normal controls. The gene expression profiles in GSE54236 [30] are based on GPL6480, including 81 HCC and 80 normal controls. The *normalizeBetweenArrays* function in the limma package [31] was used to normalize the gene expression profiles. If a gene corresponded to multiple probes, the average expression value of these probes was chosen as the expression value of the gene. Eight low-grade chronic hepatitis and 12 high-grade chronic hepatitis samples in GSE89377 and 3 cirrhotic liver tissues from patients without HCC in GSE6764 were removed from the analysis in the present study. RNA sequencing (displayed as read count) and clinical information of HCC were downloaded from The Cancer Genome Atlas (TCGA, https://www.cancer.gov/) [32]. The workflow of the present study is shown in Figure 8.

### Differentially expressed gene (DEG) analysis

The DEGs in cirrhosis and HCC samples (cirrhosis, low-grade dysplastic nodules, high-grade dysplastic nodules, and HCC) compared to the normal liver samples were screened using the limma package in R and GSE89377. The fold changes (FCs) in the expression of individual genes were calculated, and genes with |log2FC| > 1 and P < 0.05 adjusted by the false discovery rate (FDR) were considered significant.

### Weighted gene correlation network analysis (WGCNA) in GSE89377

We extracted the expression profile of DEGs in GSE89377 to perform WGCNA [33]. The phenotypes were converted to numbers for the analysis: 1 indicates normal liver, 2 indicates low-grade chronic hepatitis, 3 indicates high-grade chronic hepatitis, 4 indicates cirrhosis, 5 indicates low-grade dysplastic nodules, 6 indicates high-grade dysplastic nodules, 7 indicates early HCC, 8 indicates grade 1 HCC, 9 indicates grade 2 HCC, and 10 indicates grade 3 HCC. First, hierarchical clustering analysis was performed using the hclust function. Then, the soft thresholding power value was screened during module construction by the *pickSoftThreshold* function. Candidate power (1 to 30) was used to test the average connectivity degrees of different modules and their independence. A suitable power value was selected if the degree of independence was > 0.9. The WGCNA R package was used to construct coexpression networks (modules); the minimum module size was set to 30, and each module was assigned a unique color.

### Functional enrichment analysis

To explore the biology of the gene modules, Gene Ontology (GO) and Kyoto Encyclopedia of Genes and Genomes (KEGG) pathway enrichment analyses were performed using the clusterProfiler package [34] in R. P < 0.05 was considered significant.

### Identification of hub genes and calculation of the HGSVA score

In WGCNA, the module eigengene was the first principal component of the expression matrix for a module. The module eigengene was considered an average gene expression value for genes in a module. Phenotype was converted into a numerical value, and a regression analysis was performed between the module eigengene values and the phenotype. Module membership (MM) was defined as the association between a gene and its module, and gene significance (GS) was defined as the correlation of a gene with a phenotype. Genes with a high GS and MM were considered to be hub genes in the module. In the present study, a gene with GS > 0.7 and MM > 0.8 was considered a hub gene. Thus, multiple hub genes formed the hub gene set. Gene set variation analysis (GSVA) [35] was used to score individual samples against the hub gene set, and each sample received an HGSVA score.

### Validation of the HGSVA score, ROC curve analysis and univariate/multivariate Cox proportional hazards analyses

The HGSVA scores of all HCC samples were calculated in GSE6764, GSE49515, GSE54236 and the TCGA as in GSE89377. The GSE6764 and TCGA data sets were used to validate the increasing trend in the HGSVA score with the progression from cirrhosis to HCC. In addition, ROC curve analysis was conducted using the pROC package [36] to assess the diagnostic value of the peripheral blood HGSVA score for HCC in GSE49515. Patients with HCC in the GSE54236 and TCGA data sets were separated into high- and low-score groups based on the median HGSVA score. Kaplan-Meier survival analysis with the log-rank method was performed to explore the association of HGSVA score with recurrence-free survival (RFS) or overall survival (OS) using the survival package (https://CRAN.R-project.org/package=survival) in R. Moreover, univariate/multivariate Cox proportional hazards analyses were applied to compare the relative prognostic value of the HGSVA score with that of routine clinicopathological features in the TCGA.

### Validation of the differential expression of hub genes at the protein level

The Human Protein Atlas (https://v15.proteinatlas.org/) [37] contains information for a large majority of human protein-coding genes, including their RNA and protein expression and localization. We scanned The Human Protein Atlas web tool to validate the differential expression of the hub genes at the protein level.

### Mutation and methylation analyses

To explore the potential mechanism of the differential expression of the hub genes, we scanned these genes for mutations and methylation profiles. The TCGAbiolinks package [38] was used to download and scan the mutation status of the hub genes. In addition, we explored the DNA methylation of these genes using Wanderer (http://maplab.imppc.org/wanderer/) [39], which is an intuitive network tool that can be used to retrieve DNA methylation and gene expression data for different tumor types in TCGA databases.

## Supplementary Material

Supplementary Table 1

## References

[1] Coskun M. Hepatocellular Carcinoma in the Cirrhotic Liver: Evaluation Using Computed Tomography and Magnetic Resonance Imaging. Exp Clin Transplant. 2017 (Suppl 2); 15:36–44. 28301997 10.6002/ect.TOND16.L10

[2] Hao S, Fan P, Chen S, Tu C, Wan C. Distinct Recurrence Risk Factors for Intrahepatic Metastasis and Multicenter Occurrence After Surgery in Patients with Hepatocellular Carcinoma. J Gastrointest Surg. 2017; 21:312–20. 10.1007/s11605-016-3311-z 27815759

[3] Kou Y, Koag MC, Cheun Y, Shin A, Lee S. Application of hypoiodite-mediated aminyl radical cyclization to synthesis of solasodine acetate. Steroids. 2012; 77:1069–74. 10.1016/j.steroids.2012.05.002 22583912

[4] Kou Y, Cheun Y, Koag MC, Lee S. Synthesis of 14′,15′-dehydro-ritterazine Y via reductive and oxidative functionalizations of hecogenin acetate. Steroids. 2013; 78:304–11. 10.1016/j.steroids.2012.10.021 23238516

[5] Chen S, Cao Q, Wen W, Wang H. Targeted therapy for hepatocellular carcinoma: challenges and opportunities. Cancer Lett. 2019; 460:1–9. 10.1016/j.canlet.2019.114428 31207320

[6] Lv Y, Liang R, Hu X, Liu Z, Liao X, Lin Y, Yuan C, Liao S, Li Q, Zhang J, Li Y. Combination of oxaliplatin and S-1 versus sorafenib alone in patients with advanced hepatocellular carcinoma. Pharmazie. 2014; 69:759–63. 25985566

[7] Farazi PA, DePinho RA. Hepatocellular carcinoma pathogenesis: from genes to environment. Nat Rev Cancer. 2006; 6:674–87. 10.1038/nrc1934 16929323

[8] Erkekoglu P, Oral D, Chao MW, Kocer-Gumusel B. Hepatocellular Carcinoma and Possible Chemical and Biological Causes: A Review. J Environ Pathol Toxicol Oncol. 2017; 36:171–90. 10.1615/JEnvironPatholToxicolOncol.2017020927 29199597

[9] Budny A, Kozłowski P, Kamińska M, Jankiewicz M, Kolak A, Budny B, Budny W, Niemunis-Sawicka J, Szczypiór G, Kurniawka B, Burdan F. [Epidemiology and risk factors of hepatocellular carcinoma]. Pol Merkur Lekarski. 2017; 43:133–139. 28987047

[10] Lok AS, Seeff LB, Morgan TR, di Bisceglie AM, Sterling RK, Curto TM, Everson GT, Lindsay KL, Lee WM, Bonkovsky HL, Dienstag JL, Ghany MG, Morishima C, Goodman ZD, and HALT-C Trial Group. Incidence of hepatocellular carcinoma and associated risk factors in hepatitis C-related advanced liver disease. Gastroenterology. 2009; 136:138–48. 10.1053/j.gastro.2008.09.014 18848939 PMC3749922

[11] Niederau C, Lange S, Heintges T, Erhardt A, Buschkamp M, Hürter D, Nawrocki M, Kruska L, Hensel F, Petry W, Häussinger D. Prognosis of chronic hepatitis C: results of a large, prospective cohort study. Hepatology. 1998; 28:1687–95. 10.1002/hep.510280632 9828236

[12] Hartke J, Johnson M, Ghabril M. The diagnosis and treatment of hepatocellular carcinoma. Semin Diagn Pathol. 2017; 34:153–59. 10.1053/j.semdp.2016.12.011 28108047

[13] Li J, Gao JZ, Du JL, Huang ZX, Wei LX. Increased CDC20 expression is associated with development and progression of hepatocellular carcinoma. Int J Oncol. 2014; 45:1547–55. 10.3892/ijo.2014.2559 25069850

[14] Lin X, Yang Y, Guo Y, Liu H, Jiang J, Zheng F, Wu B. PTTG1 is involved in TNF-α-related hepatocellular carcinoma via the induction of c-myc. Cancer Med. 2019; 8:5702–15. 10.1002/cam4.2473 31385458 PMC6745867

[15] Shen Z, Yu X, Zheng Y, Lai X, Li J, Hong Y, Zhang H, Chen C, Su Z, Guo R. CDCA5 regulates proliferation in hepatocellular carcinoma and has potential as a negative prognostic marker. OncoTargets Ther. 2018; 11:891–901. 10.2147/OTT.S154754 29503564 PMC5824752

[16] Sarathi A, Palaniappan A. Novel significant stage-specific differentially expressed genes in hepatocellular carcinoma. BMC Cancer. 2019; 19:663. 10.1186/s12885-019-5838-3 31277598 PMC6612102

[17] Wong CM, Wei L, Law CT, Ho DW, Tsang FH, Au SL, Sze KM, Lee JM, Wong CC, Ng IO. Up-regulation of histone methyltransferase SETDB1 by multiple mechanisms in hepatocellular carcinoma promotes cancer metastasis. Hepatology. 2016; 63:474–87. 10.1002/hep.28304 26481868

[18] Shi L, Zhang W, Zou F, Mei L, Wu G, Teng Y. KLHL21, a novel gene that contributes to the progression of hepatocellular carcinoma. BMC Cancer. 2016; 16:815. 10.1186/s12885-016-2851-7 27769251 PMC5073891

[19] Li L, Lei Q, Zhang S, Kong L, Qin B. Screening and identification of key biomarkers in hepatocellular carcinoma: evidence from bioinformatic analysis. Oncol Rep. 2017; 38:2607–18. 10.3892/or.2017.5946 28901457 PMC5780015

[20] Li W, Cui K, Prochownik EV, Li Y. The deubiquitinase USP21 stabilizes MEK2 to promote tumor growth. Cell Death Dis. 2018; 9:482. 10.1038/s41419-018-0523-z 29706623 PMC5924753

[21] Bhattacharya S, Reddy D, Ingle A, Khade B, Gupta S. Brief Communication: Featured Article: Histone H2A mono-ubiquitination and cellular transformation are inversely related in N-nitrosodiethylamine-induced hepatocellular carcinoma. Exp Biol Med (Maywood). 2016; 241:1739–44. 10.1177/1535370216649262 27190257 PMC5027941

[22] Shi C, Huang D, Lu N, Chen D, Zhang M, Yan Y, Deng L, Lu Q, Lu H, Luo S. Aberrantly activated Gli2-KIF20A axis is crucial for growth of hepatocellular carcinoma and predicts poor prognosis. Oncotarget. 2016; 7:26206–19. 10.18632/oncotarget.8441 27036048 PMC5041975

[23] Sarasin FP, Giostra E, Hadengue A. Cost-effectiveness of screening for detection of small hepatocellular carcinoma in western patients with Child-Pugh class A cirrhosis. Am J Med. 1996; 101:422–34. 10.1016/S0002-9343(96)00197-0 8873514

[24] Arguedas MR, Chen VK, Eloubeidi MA, Fallon MB. Screening for hepatocellular carcinoma in patients with hepatitis C cirrhosis: a cost-utility analysis. Am J Gastroenterol. 2003; 98:679–90. 10.1111/j.1572-0241.2003.07327.x 12650806

[25] Köhler F, Rodríguez-Paredes M. DNA Methylation in Epidermal Differentiation, Aging, and Cancer. J Invest Dermatol. 2019. [Epub ahead of print]. 10.1016/j.jid.2019.05.011 31427190

[26] Kou Y, Koag MC, Lee S. N7 methylation alters hydrogen-bonding patterns of guanine in duplex DNA. J Am Chem Soc. 2015; 137:14067–70. 10.1021/jacs.5b10172 26517568 PMC5704973

[27] Barrett T, Wilhite SE, Ledoux P, Evangelista C, Kim IF, Tomashevsky M, Marshall KA, Phillippy KH, Sherman PM, Holko M, Yefanov A, Lee H, Zhang N, et al. NCBI GEO: archive for functional genomics data sets—update. Nucleic Acids Res. 2013; 41:D991–95. 10.1093/nar/gks1193 23193258 PMC3531084

[28] Wurmbach E, Chen YB, Khitrov G, Zhang W, Roayaie S, Schwartz M, Fiel I, Thung S, Mazzaferro V, Bruix J, Bottinger E, Friedman S, Waxman S, Llovet JM. Genome-wide molecular profiles of HCV-induced dysplasia and hepatocellular carcinoma. Hepatology. 2007; 45:938–47. 10.1002/hep.21622 17393520

[29] Shi M, Chen MS, Sekar K, Tan CK, Ooi LL, Hui KM. A blood-based three-gene signature for the non-invasive detection of early human hepatocellular carcinoma. Eur J Cancer. 2014; 50:928–36. 10.1016/j.ejca.2013.11.026 24332572

[30] Zubiete-Franco I, García-Rodríguez JL, Lopitz-Otsoa F, Serrano-Macia M, Simon J, Fernández-Tussy P, Barbier-Torres L, Fernández-Ramos D, Gutiérrez-de-Juan V, López de Davalillo S, Carlevaris O, Beguiristain Gómez A, Villa E, et al. SUMOylation regulates LKB1 localization and its oncogenic activity in liver cancer. EBioMedicine. 2019; 40:406–21. 10.1016/j.ebiom.2018.12.031 30594553 PMC6412020

[31] Ritchie ME, Phipson B, Wu D, Hu Y, Law CW, Shi W, Smyth GK. limma powers differential expression analyses for RNA-sequencing and microarray studies. Nucleic Acids Res. 2015; 43:e47. 10.1093/nar/gkv007 25605792 PMC4402510

[32] Tomczak K, Czerwińska P, Wiznerowicz M. The Cancer Genome Atlas (TCGA): an immeasurable source of knowledge. Contemp Oncol (Pozn). 2015; 19:A68–77. 10.5114/wo.2014.47136 25691825 PMC4322527

[33] Langfelder P, Horvath S. WGCNA: an R package for weighted correlation network analysis. BMC Bioinformatics. 2008; 9:559. 10.1186/1471-2105-9-559 19114008 PMC2631488

[34] Yu G, Wang LG, Han Y, He QY. clusterProfiler: an R package for comparing biological themes among gene clusters. OMICS. 2012; 16:284–87. 10.1089/omi.2011.0118 22455463 PMC3339379

[35] Hänzelmann S, Castelo R, Guinney J. GSVA: gene set variation analysis for microarray and RNA-seq data. BMC Bioinformatics. 2013; 14:7. 10.1186/1471-2105-14-7 23323831 PMC3618321

[36] Robin X, Turck N, Hainard A, Tiberti N, Lisacek F, Sanchez JC, Müller M. pROC: an open-source package for R and S+ to analyze and compare ROC curves. BMC Bioinformatics. 2011; 12:77. 10.1186/1471-2105-12-77 21414208 PMC3068975

[37] Colwill K, Gräslund S, Graslund S, and Renewable Protein Binder Working Group. A roadmap to generate renewable protein binders to the human proteome. Nat Methods. 2011; 8:551–58. 10.1038/nmeth.1607 21572409

[38] Mounir M, Lucchetta M, Silva TC, Olsen C, Bontempi G, Chen X, Noushmehr H, Colaprico A, Papaleo E. New functionalities in the TCGAbiolinks package for the study and integration of cancer data from GDC and GTEx. PLOS Comput Biol. 2019; 15:e1006701. 10.1371/journal.pcbi.1006701 30835723 PMC6420023

[39] Díez-Villanueva A, Mallona I, Peinado MA. Wanderer, an interactive viewer to explore DNA methylation and gene expression data in human cancer. Epigenetics Chromatin. 2015; 8:22. 10.1186/s13072-015-0014-8 26113876 PMC4480445

