# Supplementary material for: A twenty gene-based gene set variation score reflects the pathological progression from cirrhosis to hepatocellular carcinoma
Source: Aging (Albany NY). 2019 Dec 15;11(23):11157–69. doi: 10.18632/aging.102518 (PMC6932912; doi:10.18632/aging.102518)
Supplement: Supplementary Table 1 [file aging-11-102518-s001.pdf]

SUPPLEMENTARY TABLE

Supplementary Table 1. Immunohistochemical gene antibody.

Gene	Antibody
TOP2A	HPA006458
CDC20	CAB004525
PTTG1	HPA008890
CDCA5	HPA023691
CCNB2	HPA008873
PRC1	HPA034521
KIF20A	HPA036909
SF3B4	HPA028578
HSP90AB1	CAB005230
CCT3	HPA006543
SETDB1	HPA018142
VPS45	HPA027425
SPDL1	HPA048146
RACGAP1	HPA043912
ZNF282	HPA024374
USP21	HPA028397
PLOD3	HPA001236
